# Dysbiosis of the intestinal fungal microbiota increases lung resident group 2 innate lymphoid cells and is associated with enhanced asthma severity in mice and humans

**DOI:** 10.1186/s12931-023-02422-5

**Published:** 2023-05-31

**Authors:** Amjad N. Kanj, Theodore J. Kottom, Kyle J. Schaefbauer, Malay Choudhury, Andrew H. Limper, Joseph H. Skalski

**Affiliations:** 1 Division of Pulmonary and Critical Care Medicine, Mayo Clinic, Rochester, MN USA; 2 Thoracic Disease Research Unit, Department of Biochemistry and Molecular Biology, Mayo Clinic, Rochester, MN USA

**Keywords:** Asthma, Candida, Mycobiota, Gut, ILC2

## Abstract

**Background:**

The gut-lung axis is the concept that alterations of gut microbiota communities can influence immune function in the lungs. While studies have explored the relationship between intestinal bacterial dysbiosis and asthma development, less is understood about the impact of commensal intestinal fungi on asthma severity and control and underlying mechanisms by which this occurs.

**Methods:**

Wild-type mice were treated with Cefoperazone to deplete gut bacteria and administered *Candida albicans* or water through gavage. Mice were then sensitized to house dust mite (HDM) and their lungs were analyzed for changes in immune response. Humans with asthma were recruited and stool samples were analyzed for *Candida* abundance and associations with asthma severity and control.

**Results:**

Mice with intestinal *Candida* dysbiosis had enhanced Th2 response after airway sensitization with HDM, manifesting with greater total white cell and eosinophil counts in the airway, and total IgE concentrations in the serum. Group 2 innate lymphoid cells (ILC2) were more abundant in the lungs of mice with *Candida* gut dysbiosis, even when not sensitized to HDM, suggesting that ILC2 may be important mediators of the enhanced Th2 response. These effects occurred with no detectable increased *Candida* in the lung by culture or rtPCR suggesting gut-lung axis interactions were responsible. In humans with asthma, enhanced intestinal *Candida* burden was associated with the risk of severe asthma exacerbation in the past year, independent of systemic antibiotic and glucocorticoid use.

**Conclusions:**

*Candida* gut dysbiosis may worsen asthma control and enhance allergic airway inflammation, potentially mediated by ILC2. Further studies are necessary to examine whether microbial dysbiosis can drive difficult-to-control asthma in humans and to better understand the underlying mechanisms.

## Background

Alterations of the intestinal microbiota can profoundly impact immune function in the lung. Several animal models have been described where alterations of intestinal microbial communities enhance the severity of asthma [1, 2]. This phenomenon occurs through crosstalk between the gut and lungs, a concept called the “gut-lung axis”. It is not an infectious state but rather represents an altered gut microbial ecosystem termed “dysbiosis” when it results in negative effects [2]. Research on gut dysbiosis has largely focused on changes to the composition of bacterial communities, but in addition to bacteria, the gut contains a diverse community of fungi in both health and disease [3]. *Candida spp.* are ubiquitous commensal fungi found in the human gut where their overgrowth has been linked to alterations in the immune system and various disease states [4, 5]. *Candida albicans* is the most prevalent *Candida* species of the human microbiota [6]. Herein, we explore the effect of *C. albicans* intestinal overgrowth on allergic airway inflammation in mice and study the role of group 2 innate lymphoid cells (ILC2) as mediators of this effect. We also examine correlations between the relative abundance of *Candida spp.* in the gut of patients with asthma and specific indicators of asthma severity and poor control.

## Results

**Fig. 1:**
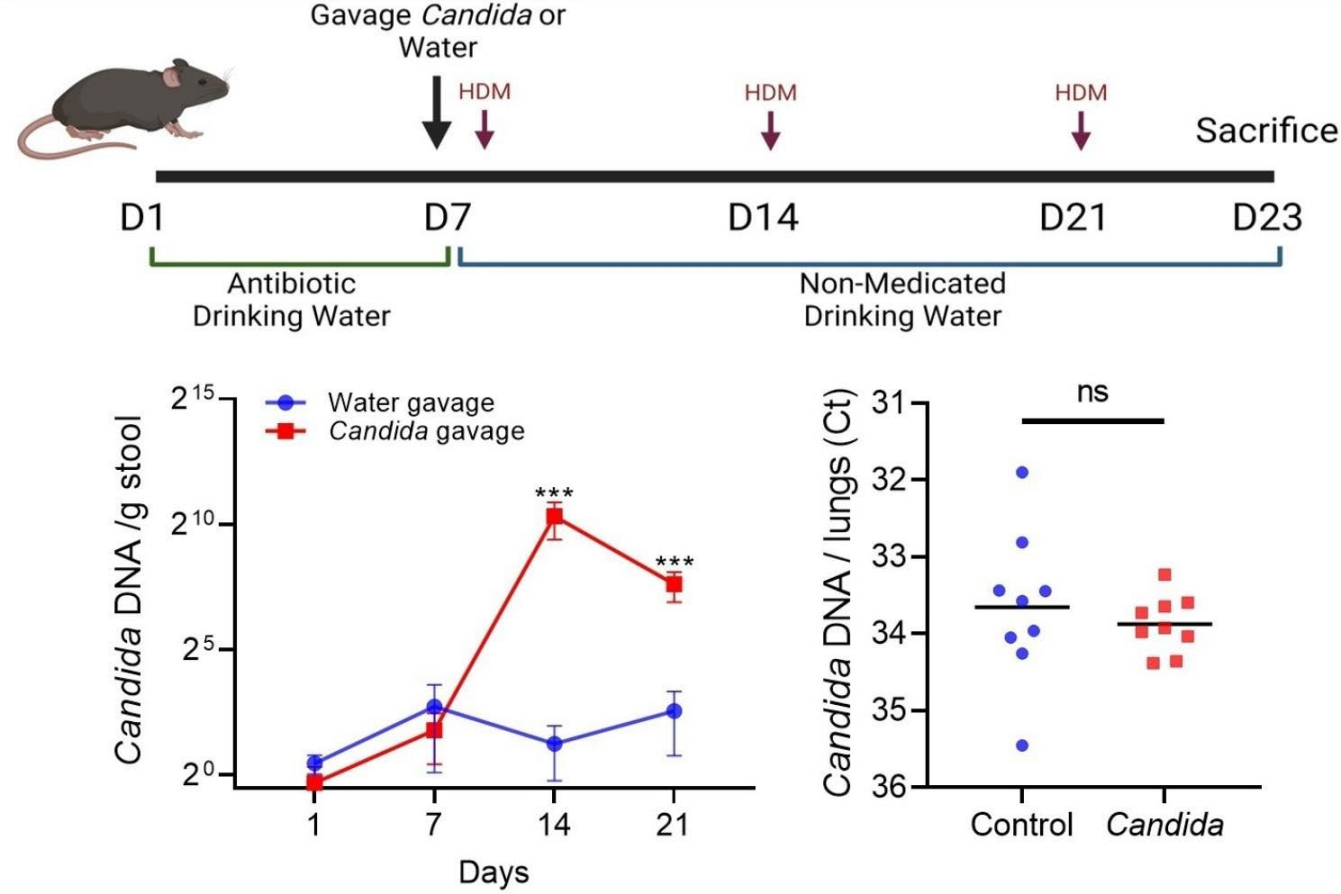
Mice were provided with cefoperazone-medicated water as the only source of water for an entire week. This was followed by a single gavage of live C. albicans or water (control), and intranasal challenge with house dust mite (HDM). The gavage of C. albicans to antibiotic depleted mice resulted in an intestinal dysbiosis state characterized by an enhanced intestinal Candida population that persisted weeks after discontinuation of the antibiotic. Mice with Candida gut dysbiosis were phenotypically normal and Candida was not detected in the lungs of mice with dysbiosis above control baseline by PCR.

**Fig. 2:**
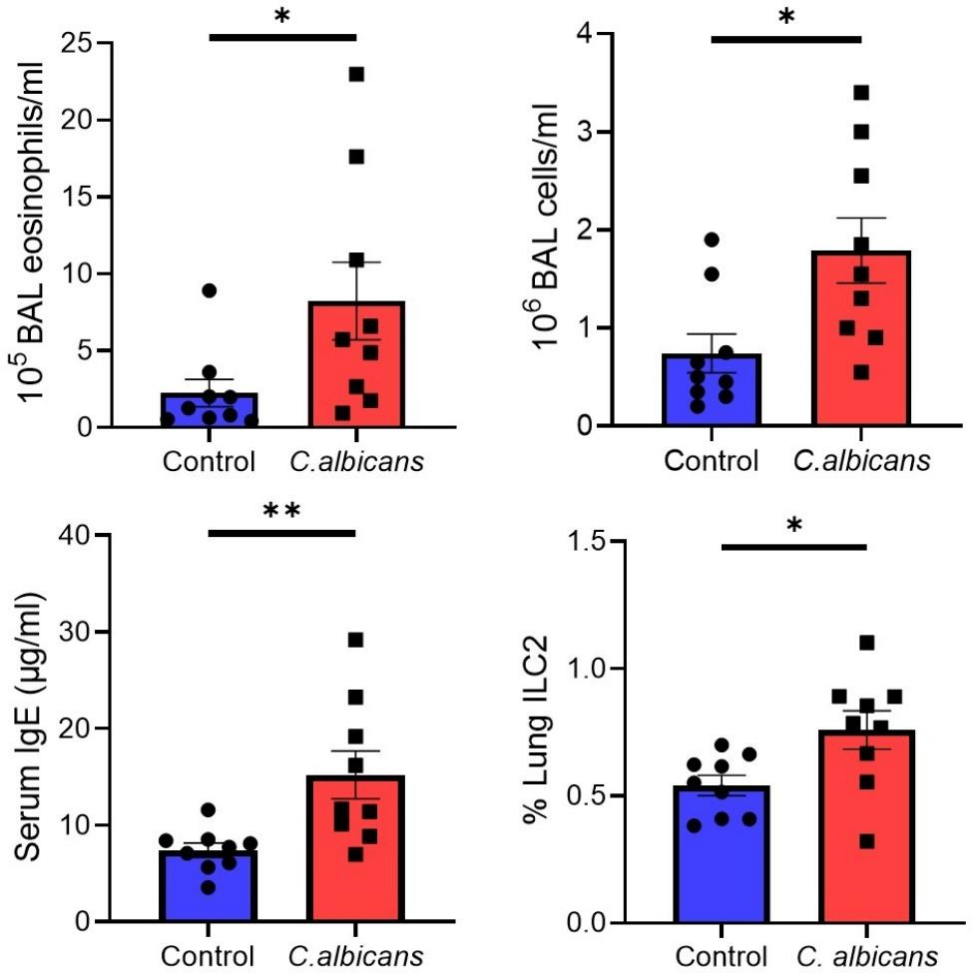
Mice with Candida intestinal dysbiosis demonstrated enhanced airway inflammation after challenge with HDM, characterized by higher total cell and eosinophil counts on bronchoalveolar lavage, higher serum total IgE and higher percent (%) of lung ILC2 cells compared to controls

All experiments followed NIH guidelines and received approval from Mayo Clinic’s Institutional Animal Care and Use Committee (#A00004926-20) and Institutional Review Board (#20-008948). We generated *Candida* gut dysbiosis using a previously described protocol of antibiotic depletion of commensal bacteria followed by gavage of live fungal organisms [1, 7]. First, we treated C57BL/6 female mice with 7 days of Cefoperazone-medicated water to deplete gut bacteria. This was followed by a one-time gavage of 10^7^ live *C. albicans* yeast or water. We then induced allergic airway inflammation by administering 50 µg of *Dermatophagoides pteronyssinus* house dust mite (HDM) intranasally every week for a total of 3 weeks. The gavage of *C. albicans* to antibiotic depleted mice resulted in a stable intestinal dysbiosis state characterized by an enhanced intestinal *C. albicans* population that persisted at least 2 weeks after discontinuation of the antibiotic (Fig. 1). Mice with *C. albicans* gut dysbiosis were phenotypically normal, but when challenged with HDM, they exhibited an enhanced Th2 response, manifesting with greater total white cell and eosinophil counts in the airway and higher total IgE concentrations in the serum (Fig. 2).

**Fig. 3:**
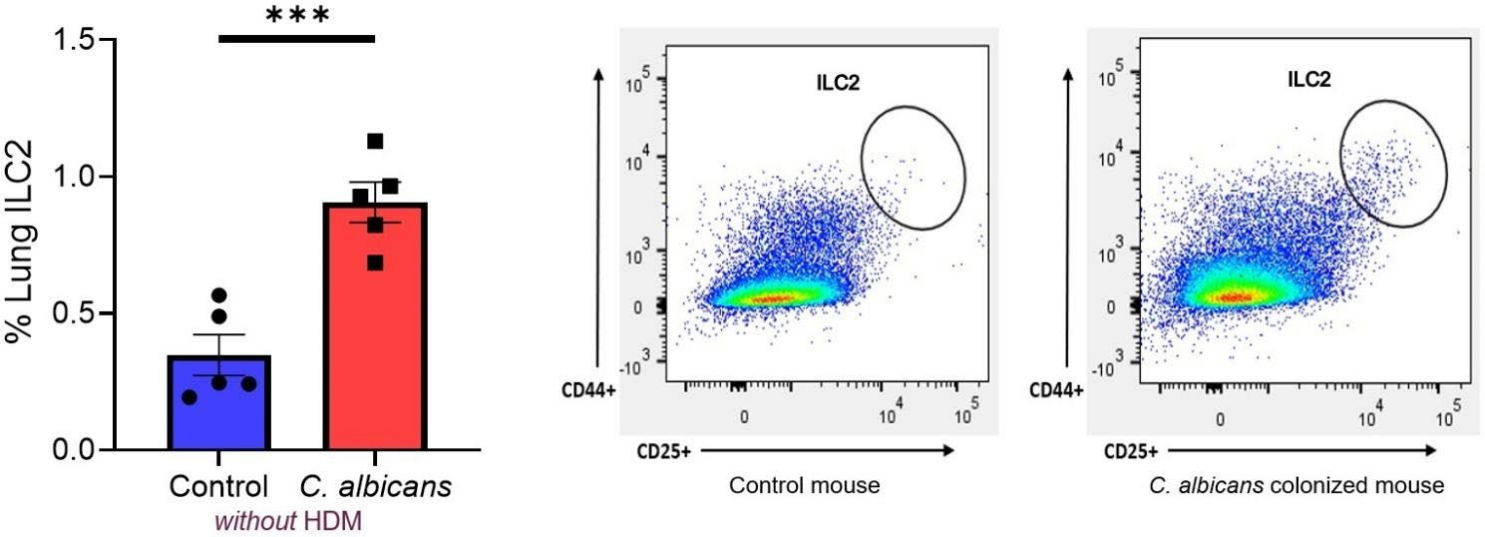
The increase in %ILC2 population in the lungs was also observed without HDM challenge suggesting ILC2 may be key mediators of observed gut-lung axis effects. ILC2 were defined as Lin- CD25 + CD44+.

We next explored the mechanisms underlying the enhanced inflammatory response observed in *C. albicans* dysbiosis mice. Enhanced lung abundance of *C. albicans* was not detectable by real-time PCR in the lungs of mice suggesting that the increased inflammation was not due to spillover of *C. albicans* into the lungs. Because *C. albicans* dysbiosis mice responded differently to HDM challenge, we hypothesized that a difference would be observed in the lung immunophenotype of *C. albicans* dysbiosis mice in the resting state, prior to challenge with HDM. To account for this enhanced inflammatory response, we considered several cell types including ILC2 which are important mediators of the type 2 inflammatory response in eosinophilic asthma [8]. ILC2 cells are native to both the gut and lung and react to fungal elements [9, 10]. We profiled the immunophenotype of *C. albicans* dysbiosis mice in the resting state prior to HDM challenge. These mice demonstrated increased abundance of lung resident ILC2 cells compared to control mice without dysbiosis which may explain the observed difference in airway inflammation following HDM challenge (Fig. 3).

**Fig. 4:**
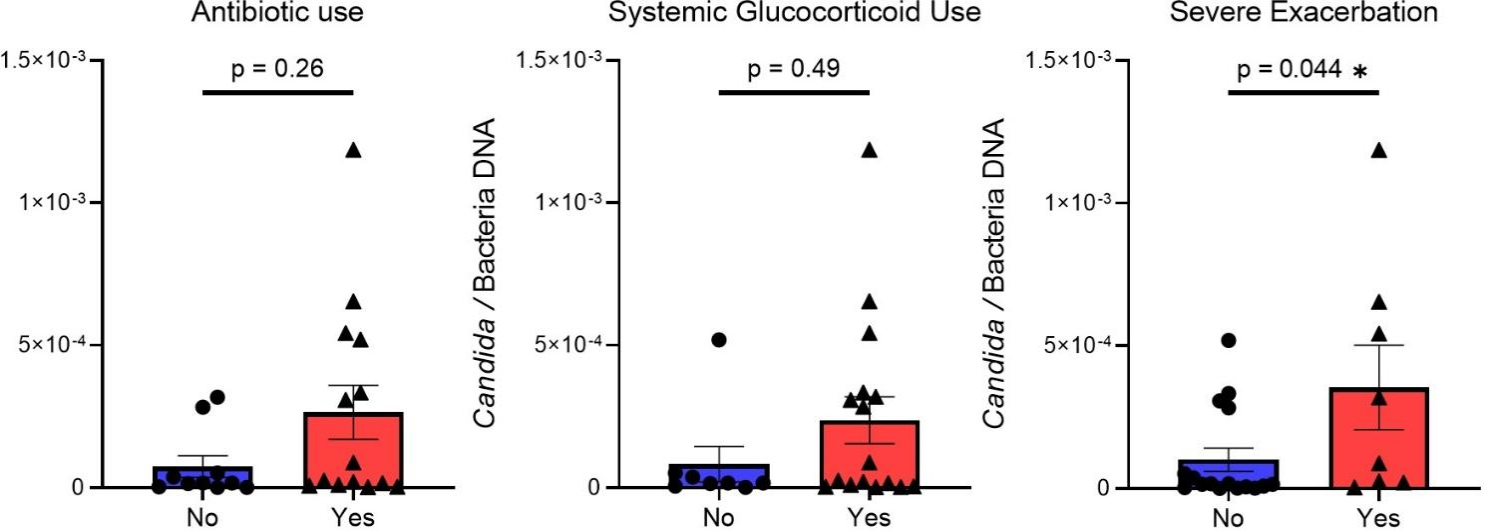
To investigate whether Candida gut dysbiosis might be observed in humans with difficult to control asthma, 24 patients with asthma were recruited. There was no significant association between either antibiotic (n = 14; 58%) or systemic glucocorticoid (n = 16; 67%) use in the past year and Candida-to-bacteria DNA in stools. Patients with severe asthma exacerbation (defined as any asthma-related emergency department visit or hospitalization) in the past year (n = 8; 33%) had a higher median Candida-to-bacteria DNA ratio in stools

We then investigated whether any of these phenomena might be observed in humans with asthma. Consecutive adult patients with asthma and no antibiotic or systemic glucocorticoid use in the last 30 days were recruited from our severe asthma outpatient practice at Mayo Clinic in Minnesota, USA. The patients (N = 24) completed a questionnaire and provided stool samples. The patients’ median (IQR) age was 57 (46–69) years; 18 (75%) were female and most were never smokers. Asthma biomarkers for each subject were abstracted from the chart if measured within past year. The study population had a median eosinophil count of 0.3 10^9^ cells/L (IQR 0.13–0.42, n = 23), median serum immunoglobulin E of 142 kU/L (IQR 64–219, n = 14), and median oral exhaled nitric oxide of 25 parts-per billion (IQR 14–41, n = 19). The median Asthma Control Test (ACT™) score was 17.5 (15.0–19.0). Microbial DNA was extracted from stool samples as previously described [1] and the abundance of *Candida spp*. was measured relative to that of bacteria (i.e., ratio of *Candida-*to-bacteria DNA) using real-time PCR pan-*Candida* primers [1, 11]. We found no associations between the relative abundance of intestinal *Candida spp*, and antibiotics use, systemic glucocorticoids use or consumption of probiotics, all in the past year (p > 0.05, Mann-Whitney U test). There was also no correlation with the ACT™ score (r = 0.24, *p* = 0.25; Spearman’s correlation). Conversely, patients with at least one severe exacerbation (defined as any asthma-related emergency department visit or hospitalization) in the past year had a higher median *Candida-*to-bacteria DNA in the gut compared to those without any severe exacerbation (20.2 vs. 1.6 × 10^− 5^, respectively, *p* = 0.044; Mann-Whitney U test) (Fig. 4).

## Discussion

Herein, we demonstrate that mice with *Candida* gut dysbiosis state, characterized by a greater abundance of *Candida spp.* relative to gut bacteria, show increased lung ILC2 abundance and enhanced eosinophilic airway inflammation in response to HDM allergen challenge. Fungi, such *as Candida spp.*, are in continuous interplay with the gut mucosa, immune cells, and other microorganisms, but little is known about how commensal fungi influence the immune system at distant sites [3]. Our experiments showed mice with intestinal *Candida* dysbiosis have increased abundance of lung resident ILC2 cells and after HDM challenge, these mice demonstrate enhanced eosinophilic airway inflammation compared to control mice without dysbiosis.

ILC2 cells are induced by the epithelial cytokines IL-25, IL-33 and thymic stromal lymphopoietin [9]. An ILC2-mediated airway allergic inflammation was observed in response to the inhaled fungal allergen *Alternaria alternata* in naïve mice. This type-2 response was significantly diminished in the absence of IL-33 receptors [12]. In the gut however, mechanisms underlying the effect of fungal dysbiosis on airway inflammation are less clear. Fungi can be complex organisms and can influence the immune homeostasis through various ways, including microbiota-derived metabolites [13]. Certain metabolites, such as bile acids derived from the microbiota following a diet of inulin fibre, were shown to trigger eosinophilia and type 2 inflammation in the lungs of mice through ILC2 [14]. Recently, Pu et al. suggested that changes to the gut microbiota promote the migration of ILC2 cells from the gut to the lung [15]. It is not entirely clear whether the increase in ILC2 population observed in the lung of mice with *Candida* gut dysbiosis was due to expansion of ILC2 cells native to the lung, or migration of ILC2 cells from the gut, or both. Nonetheless, our experiments suggest ILC2 population changes may be a mediator of the gut-lung axis enhancement of eosinophilic asthma.

We have shown that mice with *Candida* intestinal dysbiosis demonstrate an enhanced Th2 airway inflammatory response to allergen challenge. Several other intestinal dysbiosis states have been described that also enhance asthma severity in murine models including dysbiosis characterized by altered intestinal bacterial communities or overgrowth of other intestinal fungi [1, 16, 17]. This study therefore adds to a growing body of literature that dysbiosis of the intestinal microbiota can enhance asthmatic airway inflammation in murine models, but it is largely unknown whether a similar phenomenon occurs in humans with difficult-to-control asthma. We therefore performed a small pilot study of human asthma patients where we observed that enhanced intestinal *Candida* burden was associated with increased asthma-related hospital use independent of systemic antibiotic and glucocorticoid use. Though the human subject research was limited by its cross-sectional nature and small sample size, these results support the possibility that alterations of the fungal microbiome may affect asthma severity in humans similar to the observations in murine models. Further research is necessary to explore whether microbial dysbiosis may be a driver of difficult to control asthma in humans and understand the underlying mechanisms.

## Data Availability

The data generated or analyzed during the current study is available from the corresponding author on reasonable request.
